# A Pilot Study Comparing Intentional Shoulder Kinematics of Patients With Symptomatic Small-Size Supraspinatus Full-Thickness Tears With Healthy Controls

**DOI:** 10.7759/cureus.71979

**Published:** 2024-10-21

**Authors:** Nahum Rosenberg

**Affiliations:** 1 Specialists Center, National Insurance Institute - Israel, Haifa, ISR

**Keywords:** kinesiology, shoulder, supraspinatus, supraspinatus tendon tear, tendinopathy

## Abstract

Introduction: The prevailing view is that painful shoulders exhibit abnormal kinematics. This study explores the impact of symptomatic small-size supraspinatus tendon full-thickness tears on the three-dimensional (3D) kinematics of intentional, effortless shoulder movements. The hypothesis suggests that mechanical force buildup patterns, as indicated by motion jerk, will differ between healthy individuals and patients with symptomatic rotator cuff tendinopathy.

Methods: A comparative analysis was conducted on two groups: 10 patients with painful shoulders due to a Grade 1 small-size full-thickness tear of the supraspinatus tendon (Group A) and 10 healthy volunteers (Group B). Participants performed a standardized effortless shoulder movement task involving arm elevation and depression without external resistance, while an arm-attached accelerometer recorded movement data. The primary outcome was jerk (a derivative of acceleration), normalized to lean body mass (LBM), with comparisons made both within and between groups.

Results: Vertical angular velocity was consistent across the study groups and in both arms of healthy volunteers, with mean values ranging from 70 to 86 degrees per second (deg/sec) (p = 0.79). No significant differences in jerk values were observed between groups or between dominant and non-dominant limbs in Group B (mean values: 0.001 to 0.004 m/sec³/kg LBM; p > 0.05).

Conclusion: This pilot study suggests that small supraspinatus tendon full-thickness tears, when pain is the primary symptom, do not significantly affect the 3D kinematics of effortless shoulder movements. These findings challenge the notion that pain from rotator cuff tears leads to altered shoulder kinematics, with potential implications for clinical decision-making and assessing functional disability. Larger studies are needed to confirm these results.

## Introduction

The shoulder joint movement and stability are controlled by an interrelation of upper girdle muscles [1]. The shoulder has a global range of movements, including flexion, abduction, extension, adduction, and rotation. Each movement's normal range of motion is about 160-180 degrees (deg) [2]. The interrelation among shoulder girdle muscles controls these movements. The deltoid muscle is primarily responsible for arm elevation [3], the rotator cuff muscles help centralize the humeral head into the glenoid fossa and control the rotation movements [4], and the scapular stabilizing muscles, i.e., the serratus anterior and trapezius, control the scapulothoracic rhythm [5]. Understanding and optimizing these aspects of shoulder mechanics can significantly impact daily activities' efficiency, safety, and comfort. The routine daily activities that require arm elevation include reaching for objects above shoulder height, personal hygiene, recreational sports, and exercise in manual work-related tasks [3]. Daily activities that demand centralization of the humeral head and rotational control involve reaching across the body, pushing and pulling, lifting and carrying objects from the ground, and transporting them at various heights to prevent impingement or dislocation. Additionally, activities like throwing in sports require precise rotational control and stabilization of the humeral head to avoid injury [4].

The rotator cuff muscles are essential in maintaining shoulder stability and mobility while acting in unison. Normal shoulder movement involves coordinated motion of the glenohumeral joint, the scapula, and the clavicle [4]. Adequate muscle stability and proper scapulothoracic rhythm help maintain good posture, essential for all activities, including sitting, standing, and walking.

However, a symptomatic supraspinatus tendon full-thickness tear, expressed by pain, might affect the shoulder's kinematics, leading to altered movement patterns.

Rotator cuff tendinopathy typically involves microstructural changes in the tendon and its function, often due to overuse, aging, or injury. A combination of intrinsic and extrinsic factors causes this common condition. Intrinsic factors, such as tendon structure and composition changes, can be degenerative (affected by age or systemic connective tissue disorders) or following acute injury [6]. Extrinsic factors, like mechanical stress on the tendon from the adjacent bony structures (scapular glenoid and humeral head), can be due to repetitive overhead activities or poor posture, i.e., microtrauma [7]. Rotator cuff tendinopathy can lead to rotator cuff tendon tears due to impaired tendinous microstructure [6].

Rotator cuff muscles and tendon tears can be caused by repetitive overhead motions, direct trauma to the shoulder, or inflammatory conditions, such as connective tissue diseases or subacromial mechanical compromise. Shoulder impingement syndrome occurs when the rotator cuff tendons compress between the humerus and the acromion, causing pain and inflammation. Intrinsic factors include changes in the tendon structure and composition, such as collagen degeneration, calcification, and fibrosis. Extrinsic factors include mechanical stress on the tendon, such as repetitive overhead activities or poor posture [6,7]. Age-related degeneration of the rotator cuff tendons is also a common contributing factor [8].

Supraspinatus tendon full-thickness tear might cause pain and weakness in the shoulder. The intuitive opinion relates the supraspinatus tendon full-thickness tear to a reduced range of motion and slower movements in the shoulder due to pain and/or loss of motor control in the shoulder muscles [9, 10]. This suggestion is supported by the evidence that symptomatic supraspinatus tendon full-thickness tear causes altered shoulder kinematics while arm elevation, i.e., affects the scapulothoracic rhythm and, therefore, overall shoulder kinematics [11]. However, according to the electrophysiological measurements, there is somewhat contradictory previous evidence that there are no detectable mechanical deficiencies in shoulder external rotation in patients with tendinopathy due to subacromial impingement [11]. The tentative explanation for this controversy might be due to the complicity of shoulder motion control, which involves four articulations and numerous muscles with agonistic and antagonistic functions. Thus, it is difficult to estimate the normal functional motions of the shoulder and relate them to the pathological. Therefore, there is a previous experimental effort to investigate the effect of rotator cuff tendinopathy on shoulder kinematics using three-dimensional (3D) motion analysis on the theoretical models [12]. Still, these data are not sufficient for clinical evaluation. The substantial information on physiological and pathological patterns of shoulder movement is essential for understanding the kinesiology of the shoulder, with and without rotator cuff disarrangement. Such information is crucial for planning rehabilitation methods and preventing injuries and should be available at the routine clinical evaluation.

Therefore, this study aims to investigate the intentional kinesiology of the shoulder by hypothesizing that recording the 3D movement patterns of the shoulder will reveal detectable differences in the mechanical force buildup during shoulder movement between healthy individuals and patients with a full-thickness tear of the supraspinatus tendon. This point is crucial in evaluating the degree of functional shoulder disability in patients with symptomatic supraspinatus tendon full-thickness tear when pain is the main presenting symptom.

Accordingly, this study evaluates the kinematics of 3D intentional shoulder movements in normal individuals and those with symptomatic small complete rotator cuff tears. For this purpose, the measure of acceleration change (jerk) of the shoulder movement was utilized. Jerk measurements had never been mentioned in previous studies. These measurements reflect “force buildup” evaluation. The jerk measurements represent the change in arm movement acceleration. Since the acceleration of movement is proportional to the generated force, the change in acceleration represents the change in the force generated by the arm during the shoulder movement. Since this type of mechanical evaluation is easy to implement, it might be a readily available tool in everyday clinical practice.

## Materials and methods

The study compared the mechanical force buildup while effortless (without external resistance) physiologic movement of arm elevation and depression in patients with small symptomatic (painful) supraspinatus tendon tears and in asymptomatic healthy individuals.

The force buildup was expressed by a mechanical jerk measurement, which reflects the change in acceleration of arm movement.

The study was conducted between January and November 2016 at the outpatient clinics in Rambam Medical Center, Israel. 

Study group

Group A 

Inclusion criteria: A cohort of 10 consecutive patients with pain in the shoulder who were recently diagnosed as having a degenerative grade 1 (below 1 cm in diameter [13-15]) small-size full-thickness tear in the supraspinatus tendon according to the sonographic or magnetic resonance imaging (MRI) evaluation and received no treatment and suffered from a moderate level of shoulder pain, equivalent to Grade 4-5 on the visual analog scale (VAS) with a full range of active shoulder movements. were included in the study.

Exclusion criteria: Patients with severe shoulder pain (equivalent to above Grade 6 on VAS) or with very mild shoulder pain (below Grade 4 on VAS), patients with restricted range of active shoulder movements, patients with inflammatory arthritis, diabetes mellitus, or any kind of neurological disease or following trauma to the shoulder were excluded.

Group B

Inclusion criteria: A cohort of 10 healthy volunteers with no history or complaints of any disability in shoulders having a full range of active shoulder movements were included.

Exclusion criteria: Patients with previous major trauma, occupations (professional or sports) involving repetitive upper limb efforts, and restricted painless range of active shoulder movements were excluded.

Group A consisted of seven men and three women, with a mean age of 47 years old (ranging from 21 to 64 years old). Group B comprised seven men and three women, with a mean age of 60 years old (ranging from 30 to 83 years old). There was no significant difference in age distribution between study groups (p = 0.059, t-test).

The study was conducted according to the Declaration of Helsinki guidelines and approved by the Institutional Ethics Committee of Rambam Medical Center, Haifa, Israel (protocol code and approval number: 1759, year: 2015). All the tested individuals signed the informed consent form.

Test procedure

The test procedure aimed to reflect the normal functional elevation and depression of the arm. The evaluation was of a natural, effortless, intentional shoulder movement without restriction by any external force apart from gravity. The tested individual had an accelerometer attached to the arm (resolution 0.039227 m/sec², frequency range 58.433-2016.31 Hz, mean 402 ± 22 Hz; InvenSense Inc., San Jose, CA, USA). It was positioned 10 cm distal to the lateral edge of the scapular acromion. The shoulder was adducted and in contact with the thorax, the elbow was flexed at 90°, and the forearm was in a neutral position. The individual was instructed to place the hand behind their occiput and then return it to the initial position through an active, natural movement without excessive effort. This shoulder movement engages abduction, forward flexion, and external rotation during arm elevation. Conversely, during arm depression, the shoulder performs adduction and internal rotation. These testing maneuvers allow for the assessment of physiological shoulder movement within a 3D plane. The measurements of vertical angular velocity and acceleration of arm movements in three dimensions were recorded numerically and transferred by Bluetooth® short-range wireless connection to a computer.

In Group B, measurements were taken on both arms, whereas in Group A, measurements were conducted only on the arm with rotator cuff pathology.

Data analysis and statistical analysis

The size of the study groups was planned according to the 0.7 minimal power of the statistical comparison (based on the expected 20% difference of means, 15% standard deviation, and an alpha value of 0.05) and calculated by using SigmaStat software for Windows, version 2 (SPSS Inc., Chicago, Il, USA). Accordingly, 10 consecutive individuals from two study groups each were examined.

**Figure 1:**
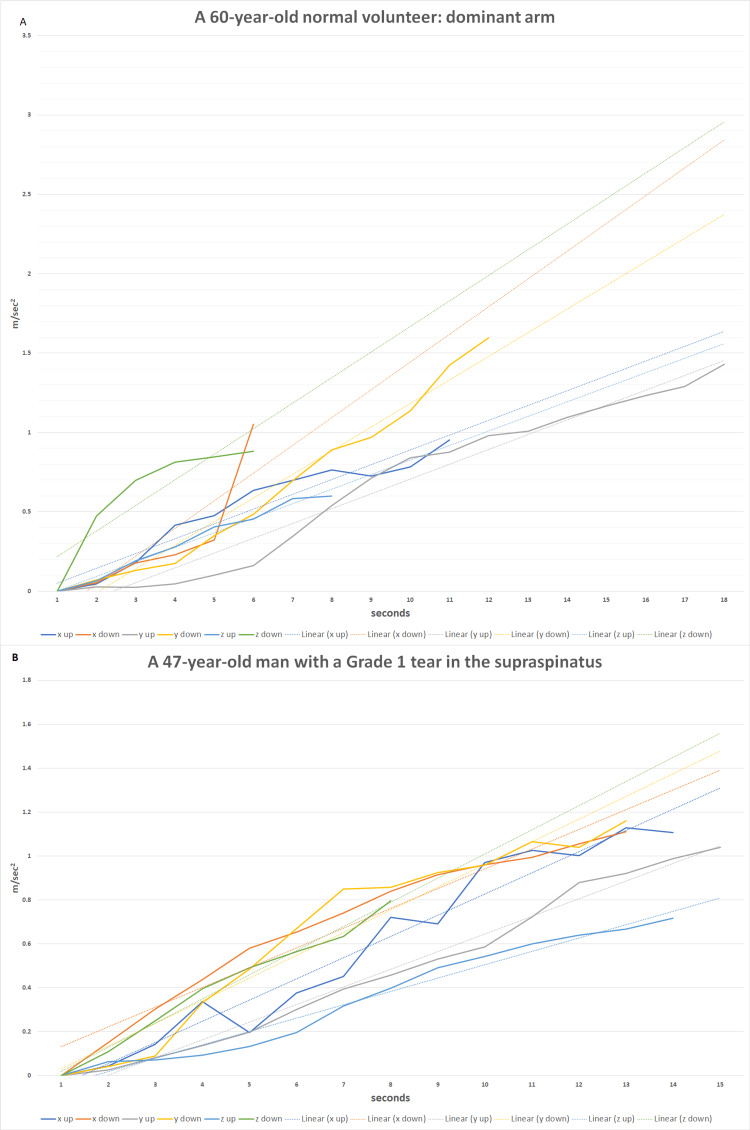
Representative examples of the acceleration profiles (not normalized) during shoulder standardized movement Dashed lines represent linear trendlines: (A) recording in the normal shoulder (Group B); (B) recording in the shoulder with supraspinatus tendon tear (Group A) x, z: horizontal axes of movement; y: vertical axis of movement; up: arm elevation; down: arm depression

The plotted arm acceleration values were processed to represent the linear trendline. A linear trendline slope was calculated using a simple linear regression method [16] using Microsoft Excel software (Microsoft® Excel® for Microsoft 365 MSO, Version 2303 Build 16.0.16227.20202, 64-bit, Microsoft Corp., Redmond, WA). The linear trendline slope represents the mean jerk, a change of acceleration (a derivative of acceleration), during the shoulder movement. The evaluation of the mean jerk as the indicator of force buildup during shoulder movement standardizes the measured acceleration profiles (Figure 1).

The mean jerk values were normalized to the lean body mass (LBM) to eliminate the effect of the variations in the overall fitness conditions of the examined subjects. The LBM was calculated using the following formula [17]:

LBM(men)=(1.10×Weight(kg))-128×(Weight(kg)2/(100×Height(m)2) LBM (women)=(1.07×Weight((kg))-148×( Weight(kg)2/(100×Height(m)2)

For ease of representation, the movements in the negative direction on the 3D axes were converted into positive values.

When the normal distribution of values was confirmed, a paired t-test was used to compare jerk values in normal volunteers' dominant and nondominant upper limbs. Otherwise, the signed rank test was implemented.

For comparison of jerk values in dominant and non-dominant upper limbs in normal volunteers and in patients with rotator cuff pathology, one-way ANOVA was used when the normal distribution of values was confirmed; otherwise, Kruskal-Wallis one-way ANOVA on ranks was implemented (by SigmaStat software for Windows, version 2).

## Results

Both groups had similar gender distribution and had no significant difference in age distribution (p = 0.06, t-test).

**Figure 2:**
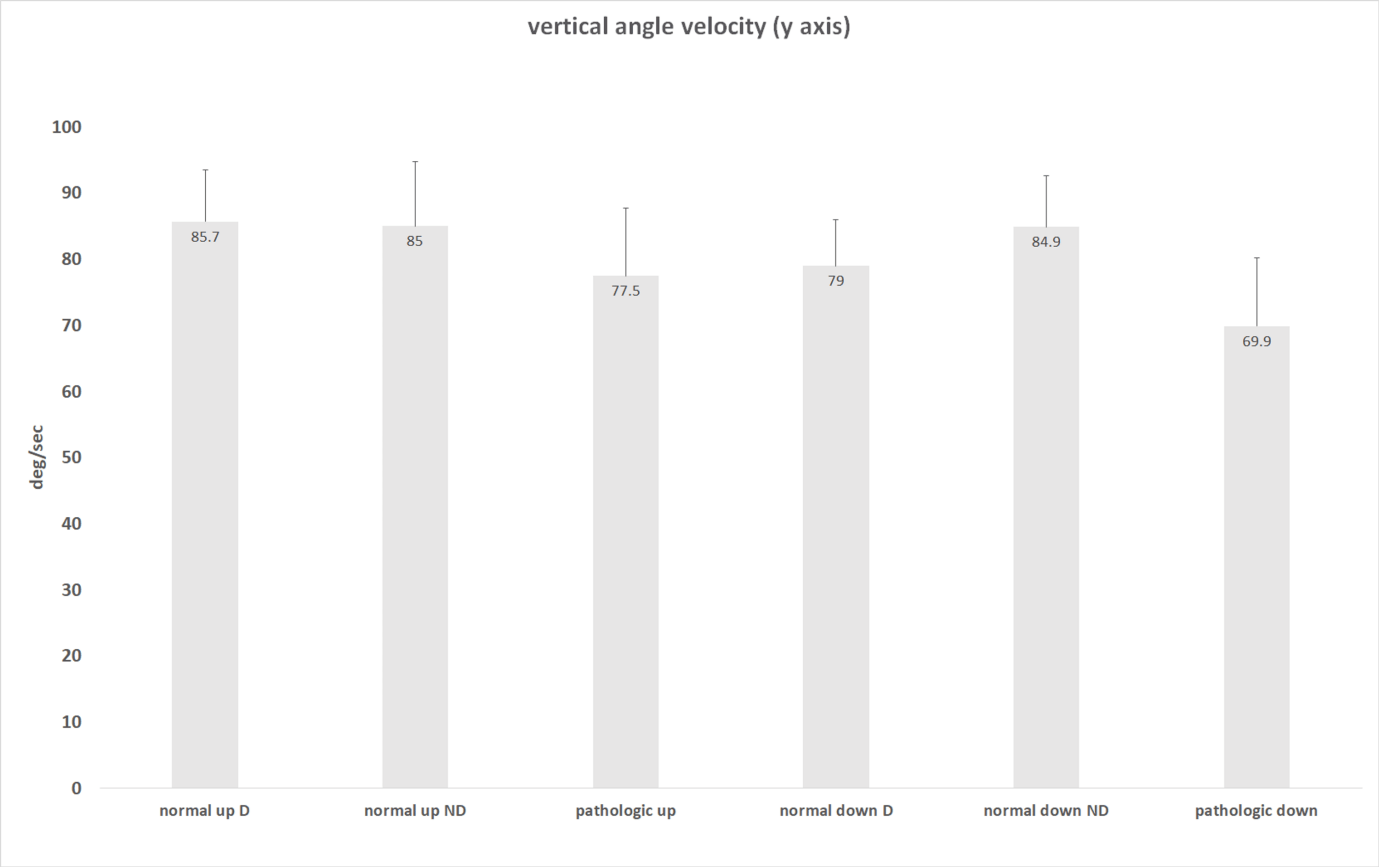
Vertical angular velocity of shoulder movements Mean values are presented. The vertical bars represent the standard error of means (SEM). There was no significant difference among the study groups (p=0.79, one-way ANOVA). D: dominant arm in normal volunteers (Group B); ND: non-dominant arm in normal volunteers (Group B); pathologic: shoulder with rotator cuff (RC) tear (Group A); up: arm elevation; down: arm depression

The vertical angular velocity, representing arm elevation and depression in the vertical axis, was similar among study groups and in both arms in the normal volunteers (in arm elevation - mean 85.7±7.8 standard error of means (SEM) deg/seconds (sec) in the dominant arm in Group B, mean 85.0±9.8 SEM deg/sec in the non-dominant arm in Group B, mean 77.5±10.2 SEM deg/sec in Group A; in arm depression - mean 79.0±6.0 SEM deg/sec in the dominant arm, mean 84.9±7.7 SEM deg/sec in the non-dominant arm in Group B, mean 69.9±10.3 SEM deg/sec in Group A; the range of mean values was 69.9-85.7 deg/sec (p=0.79, one-way ANOVA) (Figure 2).

**Figure 3:**
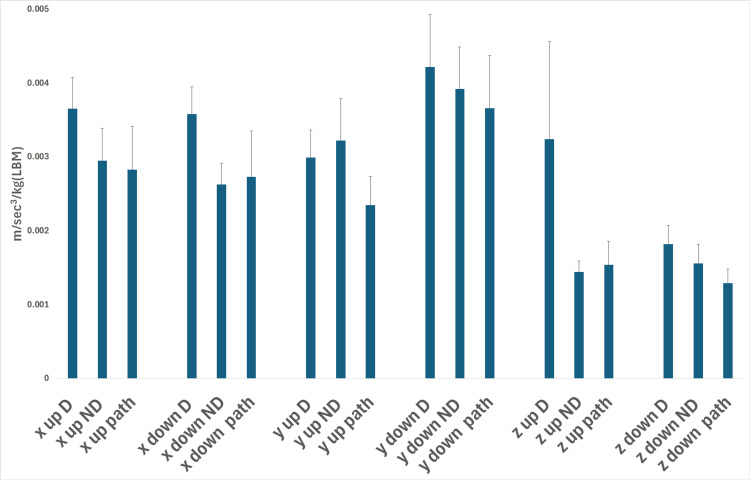
Mean jerk of shoulder voluntary movement normalized to lean body mass (LBM) The vertical bars represent the standard error of means (SEM). There was no significant difference between the study groups (Groups A and B) and between the individual normal volunteer shoulders in Group B (p>0. 05, one-way ANOVA, Kruskal-Wallis, paired t-test, respectively). D: dominant arm in normal volunteers (Group B); ND: non-dominant arm in normal volunteers (Group B); path (Group A): shoulder with rotator cuff tear; x, z: horizontal axes of movement; y: vertical axis of movement; up: arm elevation; down: arm depression

In all three dimensions, the mean values of the jerk of shoulder intentional movements normalized to the LBM were not significantly different between Groups A and B for both shoulders (mean values ranged from 0.001 to 0.004 m/sec3/kg (LBM) (p>0.05, one-way ANOVA and Kruskal-Wallis) (Figure 3).

The comparison of the normalized to the LBM values of the jerk of shoulder intentional movements showed no difference between the dominant and nondominant upper limb for every individual in Group B (p > 0.05, paired t-test and signed rank test (Figure 3).

## Discussion

The normal range of motion, shoulder rhythm, glenohumeral joint stability, shoulder muscle activation, and other normal shoulder dynamics are important factors in diagnosing and treating shoulder problems. Therefore, understanding normal shoulder kinesiology is essential for diagnosing and treating shoulder pathologies.

The current study addresses the effect of the supraspinatus tendon small full-thickness tear, expressed by moderate shoulder pain, on shoulder kinesiology.

The study protocol investigated the intentional physiological effortless shoulder movement (arm elevation and depression movements without external resistance), which appeared unaffected by potential variations of the shoulder angular velocity during intentional efforts.

Two demographically similar groups of individuals were investigated (same gender distribution and no significant difference in age distribution between the groups). There was no significant difference in the mean angular velocities of the shoulder movements among the study groups. Therefore, this type of study protocol excludes the requirement of biofeedback techniques for the meaningful comparison between the study groups.

There is an intuitive opinion that shoulder kinesiology depends on age, sex, and other individual factors. Certain conditions, such as rotator cuff tendinopathy, might also affect shoulder kinesiology and alter movement patterns due to altered shoulder kinematics, i.e., abnormal arm elevation and altered scapulothoracic and glenohumeral rhythms, which subsequentially can lead to compensatory movements, such as increased scapular elevation or anterior tilting, to reduce pain or compensate for weakness. In the symptomatic supraspinatus tendon full-thickness tear, delayed activation, or reduced force generation in the supraspinatus may cause other muscles to compensate, leading to imbalance and further dysfunction as part of the expected muscle activation patterns’ alteration [18]. Therefore, the intuitive general opinion indicates that tendinopathy-related pain can alter proprioception, leading to suboptimal neuromuscular control of the shoulder. This can exacerbate instability and abnormal movement patterns that might contribute to the secondary subacromial impingement.

The current report, however, does not fully support this opinion. Specifically, the report reveals that intrinsic moderate supraspinatus tendinopathy expressed by supraspinatus tendon full-thickness tear does not significantly affect the 3D intentional shoulder kinematics, which includes the mechanical force buildup necessary for shoulder movement. This type of tendinopathy, where pain is the primary clinical expression, does not seem to interfere with effortless shoulder mechanics. This finding challenges the conventional belief that intrinsic shoulder tendinopathies, such as those involving the supraspinatus tendon, would substantially impact the basic shoulder function and, by extension, the patient's overall disability.

Supraspinatus tendon full-thickness tear is a common condition characterized by pain and dysfunction in the shoulder. The supraspinatus tendon is a crucial component of the rotator cuff, which plays a vital role in shoulder stability and movement. It has been traditionally believed that a supraspinatus tendon full-thickness tear could lead to significant impairments in shoulder function, thus contributing to a patient's disability [19]. However, this report suggests otherwise, indicating that the mechanical aspects of shoulder effortless movement remain largely intact despite the presence of a supraspinatus tendon full-thickness tear.

This unexpected outcome holds significant implications for how we evaluate the disability of patients with supraspinatus tendon full-thickness tear. Typically, the evaluation of shoulder disabilities has placed a considerable emphasis on the presence of pain and its assumed impact on shoulder mechanics. However, the report’s findings suggest that the presence of pain of moderate level of severity, while clinically significant, does not necessarily correlate with a mechanical deficit in shoulder kinematics.

Shoulder movement involves a complex interplay of muscles, tendons, and joints, with the supraspinatus playing a crucial role in the abduction and stabilization of the shoulder joint. The mechanical force buildup in the shoulder during movement is a critical factor in maintaining this complex kinematic chain. If a small-size supraspinatus tendon full-thickness tear does not significantly disrupt this force buildup, it suggests that the shoulder's compensatory mechanisms might be more robust than previously thought.

Furthermore, the finding that limb dominancy does not influence the effortless force buildup in normal shoulders adds another layer of complexity to evaluating shoulder disabilities. Limb dominance has often been considered a factor in assessing the severity of shoulder conditions under the assumption that the rotator cuff pathology in the dominant limb would express a higher degree of disability. However, the report indicates this is not necessarily the case, as normal kinematics do not differ between dominant and non-dominant limbs.

This insight should prompt a reevaluation of current clinical practices and disability assessment criteria for patients with small-size supraspinatus tendon full-thickness tears. It suggests that clinicians might need to place greater emphasis on functional assessments and less on the presence of pain alone when determining the extent of disability. Additionally, rehabilitation strategies might need to be adjusted to focus more on maintaining and enhancing shoulder kinematics rather than solely addressing pain [20].

This report provides a nuanced perspective on the impact of intrinsic moderate supraspinatus tendinopathy, expressed by a small-size supraspinatus tendon full-thickness tear, on shoulder kinematics. By revealing that mechanical force buildup during shoulder effortless movement remains largely unaffected, it challenges traditional notions about the relationship between tendinopathy, pain, and disability. This finding underscores the need for a more comprehensive approach to evaluating and treating shoulder disabilities, considering the complexity of shoulder mechanics and the potential for compensatory mechanisms to mitigate the functional impact of supraspinatus tendon full-thickness tear. Moreover, the high diagnostic accuracy of imaging techniques in characterizing rotator cuff disorders [21] further underscores the complexity of correlating clinical findings with mechanical impairment.

Several potential reasons could explain the lack of difference in force buildup between Group A (with supraspinatus tendon full-thickness tear) and Group B (without tendinopathy). Individuals with supraspinatus tendon full-thickness tears may develop compensatory strategies to maintain normal shoulder kinematics during effortless movements. These compensatory mechanisms, such as increased activation of surrounding muscles (e.g., the deltoid or scapular stabilizers), could mask the effects of the supraspinatus tendon full-thickness tear, resulting in no observable difference in jerk motions. The study focuses on individuals with moderate tendinopathy, expressed by supraspinatus tendon full-thickness tear. The pathology might not be severe enough to impact shoulder mechanics significantly during low-demand tasks at this level. The mechanical disruption may only become apparent under higher physical stress or fatigue conditions that were not assessed in the study. Pain due to the supraspinatus tendon full-thickness tear may lead to altered neuromuscular control. Still, in moderate cases, this adaptation could stabilize the joint effectively enough to prevent differences in jerk motions during controlled, effortless movements. Pain could also lead to cautious movement patterns that minimize the appearance of abnormal kinematics. There is a possibility that some individuals in Group B, who were classified as having "normal" shoulders, might have had subclinical or asymptomatic supraspinatus tendon full-thickness tear or other minor shoulder pathologies that were not detected. This could blur the distinctions between the groups, reducing the observed differences in jerk motions. The methods used to measure jerk may not have been sensitive enough to detect subtle differences in shoulder kinematics. The study's design might not have included sufficiently challenging movements or conditions that could have amplified potential differences between the groups. Human biomechanics can be highly variable, even among healthy individuals. This natural variability might overshadow the specific effects of symptomatic supraspinatus tendon full-thickness tear on shoulder kinematics, making it difficult to detect differences between the two groups in a small sample size. Since the study focused on effortless shoulder movements, the lack of difference in jerk might indicate that the supraspinatus tendon full-thickness tear primarily affects more strenuous or complex movements that were not part of the study protocol. Thus, the supraspinatus tendon full-thickness tear may not interfere with the basic, low-effort movements assessed in this study. Understanding these factors can help refine future studies to better explore the relationship between supraspinatus tendon full-thickness tear and shoulder kinematics, potentially by including a broader range of tasks, increasing measurement sensitivity, or considering more challenging movement scenarios.

Despite the insightful findings of the current study on the effects of intrinsic moderate supraspinatus tendinopathy, expressed by supraspinatus tendon full-thickness tear, on shoulder kinematics, several limitations warrant consideration. First, the study focused exclusively on effortless shoulder movements without external resistance, which may not capture the full spectrum of functional activities that place varying demands on the shoulder complex. Second, while the demographic variables such as age and gender were matched between groups, other factors like occupational activities, athletic participation, or previous shoulder injuries were not accounted for, potentially introducing confounding variables. Third, the study did not include assessments of asymptomatic shoulders in patients with unilateral supraspinatus tendon full-thickness tear, limiting the ability to discern bilateral compensatory mechanisms or subclinical pathologies. Additionally, the reliance on imaging techniques to characterize rotator cuff disorders, although diagnostically accurate, may not fully correlate with functional impairments experienced by patients. Lastly, the study's cross-sectional design precludes establishing causal relationships or observing the progression of supraspinatus tendon full-thickness tear and its long-term effects on shoulder mechanics. Future research on large-size study groups incorporating a broader range of functional tasks, comprehensive participant histories, bilateral assessments, and longitudinal designs would provide a more exhaustive understanding of shoulder kinematics in the context of rotator cuff tendinopathies.

## Conclusions

The data presented should have an important indicative impact on evaluating the disability of patients with moderate rotator cuff tendinopathy with small supraspinatus tendon full-thickness tear. It might influence the decision-making regarding treating these patients, i.e., the indication for surgical vs. conservative treatment. The evaluation of the extent of physical disability might also be affected by this observation. However, it is expected to be different in the shoulder kinematics with increased muscular effort; as previously shown, reduced maximal isometric force is generated in arm elevation in patients with supraspinatus tendinopathy. This suggestion should be further evaluated in future studies.

Future research could utilize the presented, easily available evaluation method on shoulder kinematics under low-effort conditions as a benchmark for assessing patients with painful shoulders due to supraspinatus tendon full-thickness tear during higher-effort activities. This might enable more effective treatment decisions, advance the understanding and treatment of rotator cuff tendinopathies, and determine the level of disability in these patients.
